# Safety and efficacy of ultrasound-guided pars plana vitrectomy: a prospective, single-arm study

**DOI:** 10.1186/s40942-025-00723-z

**Published:** 2025-08-21

**Authors:** Raul Velez-Montoya, Manuela Franco-Sanchez, Katherin M. Ureña-Tejada, Ramses Rosales-Díaz, Gerardo Pedraza-Rivera, Xiadani L. De la Rosa-Gonzalez, Catalina Becerra-Revollo, Mariana Mayorquin-Ruiz, Jans Fromow-Guerra, David Berrones

**Affiliations:** 1 Retina Department, Asociación para Evitar le Ceguera en México IAP., Vicente García Torres #46. Col: San Lucas Coyoacán, México City, 04030 México; 2 Ultrasound Department, Asociación para Evitar le Ceguera en México IAP., Vicente García Torres #46. Col: San Lucas Coyoacán, 04030 México City, México

**Keywords:** Alternative, Endoscopic, Feasibility, Guided, Keratoprosthesis, Safety, Surgical technique, Ultrasound, Vitrectomy

## Abstract

**Purpose:**

To estimate the amount of eliminated vitreous after ultrasound-guided pars plana vitrectomy and complete the safety profile of the technique.

**Methods:**

We enrolled patients with vitreous hemorrhage and clear media who had an ultrasound-guided pars plana vitrectomy. The amount of eliminated vitreous was calculated by digital analysis (ImageJ) of before and after photos, obtained intraoperatively. All cases were completed under direct visualization for safety evaluation. All patients had a comprehensive ophthalmological examination at baseline and at day 1, and 1 month follow-up. Adverse events were reported in proportions ± 95%CI. A learning curve was plotted via the formula *y = ax^b.* Interobserver agreement was assessed with a Cohen-Kappa test.

**Results:**

We enrolled 62 patients. Mean age: 64.3 ± 13.3 years. Digital image analysis showed that there was a significant reduction in the number of pixels representing vitreous hemorrhage (≈ 70%, *p* < 0.01). The minimum number of cases needed to achieve a 70% or greater elimination of vitreous was 18. The proportion of potentially related adverse events to the surgical technique was 1.6%, 95%CI: 0.04–8.66).

**Conclusion:**

Ultrasound-guided pars plana vitrectomy is feasible and has an acceptable safety profile for cases with minimal to no visibility of the posterior pole.

**Supplementary Information:**

The online version contains supplementary material available at 10.1186/s40942-025-00723-z.

## Summary statement

Ultrasound-guided pars plana vitrectomy does not require optical instruments to achieve a 70% elimination of the vitreous, maintaining a similar safety profile to regular vitrectomy. The technique could be considered a viable option in urgent cases where access to a temporary keratoprosthesis or ophthalmic endoscope is not possible.

## Introduction

A safe and effective pars plana vitrectomy (PPV) needs three key elements: (1) A properly trained surgeon (skills); (2) Surgical technology (vitrectomy machine/surgical microscope/surgical adjuvants); and (3) A reasonably clear cornea that allows direct visualization of intraocular maneuvers [1–3]. The latter is considered the limiting factor because, even with a skilled surgeon and the right surgical equipment, the impossibility of observing our movements inside the eye significantly decreases the safety of the procedure and jeopardizes the achievement of the surgical goals [1, 4]. 

In urgent situations where even a partial removal of vitreous could potentially improve the patient’s outcome, the presence of an unavoidable corneal opacity may force the retina surgeon to choose between deferring the case or performing a blind procedure [3, 5–8]. In the latter, although feasible, the extent of vitreous removal can only be estimated based on the surgeon’s experience. Moreover, to minimize the risk of iatrogenic damage to intraocular structures, the range of movements of surgical instruments must be limited to the central vitreous cavity, which further decreases the effectiveness of the vitreous removal [4]. 

In a previous manuscript, our group described an alternative method to maximize the amount of vitreous removed while improving the overall safety of a blind procedure [4]. This novel surgical technique (ultrasound-guided pars plana vitrectomy or USG-PPV) uses an ophthalmic ultrasound to guide the vitrectomy cutter movements inside the vitreous cavity [4]. Our initial experience with this technique suggested that eliminating the vitreous while maintaining proper visualization of the cutter and anatomical landmarks in real time was feasible. However, a proper assessment of the efficacy of the technique and its safety profile was still pending [4]. 

In the current manuscript, we aim to expand our experience with this technique in a controlled environment, and include a full assessment of the safety profile, a precise quantification of the amount of vitreous removed through digital image analysis, and estimation of the learning curve. For this purpose, we performed USG-PPVs during the first minutes of scheduled PPV surgeries for vitreous hemorrhages in patients with clear media.

## Methods

This was a prospective, investigational, nonrandomized clinical trial approved by the internal review board of the *Asociación para Evitar la Ceguera IAP* (17 CI 09 003 142 RE-20-22). The study was conducted according to the tenets of the Declaration of Helsinki and Good Clinical Practice guidelines. All sensitive data were managed according to the Mexican Federal Law for the Protection of Personal Data in Possession of Individuals (NOM-024-SSA3-2010) and the Health Insurance Portability and Accountability Act (HIPAA) rules. All participants signed an informed consent form before enrollment. No generative artificial intelligence software was used to create or correct the text of this manuscript.

We enrolled patients who were 18 years of age or older, had clinical diagnoses of grade III or higher vitreous hemorrhage secondary to diabetic retinopathy, had no significant corneal opacities, and who were scheduled to undergo a combined procedure of phacoemulsification with IOL implantation and PPV. We excluded patients with vitreous hemorrhages suspected to be secondary to diagnoses other than diabetic retinopathy, patients with a previous history of corneal diseases or corneal decompensation, macular edema, age-related macular degeneration, inflammatory eye diseases, glaucoma, pseudoexfoliation, rubeosis iridis and/or evidence of any degree of retinal traction on a b-scan ultrasound performed during the screening visit.

We choose to enroll patients with vitreous hemorrhage and clear media and perform a USG-PPV during the first minutes of their scheduled procedure due to the following reasons: the absence of significant proliferations provided an undistorted anatomy of the posterior pole, facilitating accurate quantification of eliminated vitreous through digital image analysis and minimize the risk of iatrogenic retinal damage. Additionally, a clear cornea allowed for immediate evaluation of the posterior pole following USG-PPV within the same surgical procedure, ensuring the surgeon could address any complications, remove residual vitreous, and perform panretinal photocoagulation to complete the case.

At baseline, all patients underwent a comprehensive ophthalmological examination, which included assessment of best-corrected visual acuity (BCVA), slit lamp examination, fundus examination, intraocular pressure measurement, and b-scan ultrasound. The ophthalmologic examination was repeated the day after the surgery and at 1 month of follow-up.

**Fig. 1:**
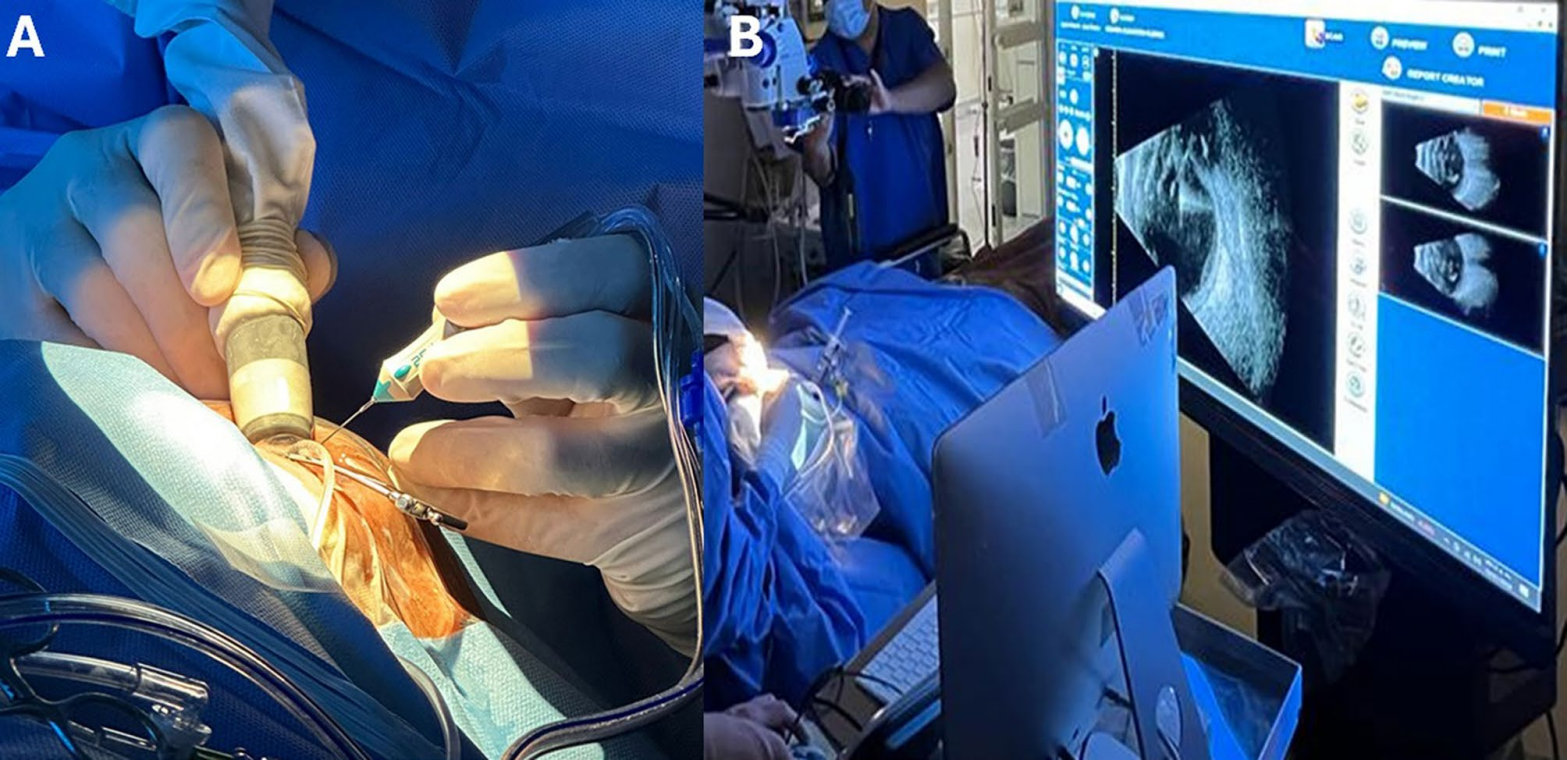
General layout of the surgical field and head-up display. **A** shows the cutter probe and the ultrasound handpiece being used simultaneously. **B** shows the ultrasound image being displayed on the head-up display, using its picture-in-picture feature

The surgical technique was described elsewhere [4]. The same surgeon (RVM) performed all surgeries. In summary, after phacoemulsification and IOL implantation, we used a standard 3-port 25-gauge pars plana vitrectomy (Constellation, Alcon Fort Worth, US) approach with active intraocular pressure control enabled, a digital-assisted display (Ngenuity, Alcon Fort Worth, US), and picture-in-picture feature. Real-time 10 MHz b-scan ultrasound was captured (Aviso, Quantel Medical, Fr; Axial Resolution: 150 mm, Lateral resolution:300 mm, Frame Rate: 16 Hz) (Fig. 1). The latter was connected directly to the digital-assisted display computer, allowing both images to be displayed on the screen in front of the surgeon and easily switched between. The vitrectomy machine parameters were the same in all cases: cut rate: 7500 cuts per minute. Vacuum: 450 mmHg. The patient’s intraocular pressure was set to 28 mmHg, with active intraocular pressure control.

After the placement of the trocars in the usual fashion and location, three baseline still frames of the posterior pole under direct visualization (Resight Fundus Viewing System, Carl Zeiss Vision Inc. KY. USA) were taken directly from the screen display, using the following parameters: the eye was in a neutral position with the center of the cornea center in the center of the image, 30% diaphragm aperture, 0.4x magnification, and 50% illumination. Then, the surgical microscope and posterior visualization system were set aside, and the image displayed on the screen of the digital-assisted display was switched to the one generated by the ultrasound equipment (supplemental video 1). The cutter movements and vitreous hemorrhage removal were guided by sequential longitudinal b-scans on each quadrant of the eye until no further movement of the vitreous was observed, with an image gain of 100–110 dB (supplemental video 2). The ultrasound probe was held in the surgeon’s non-dominant hand and firmly positioned over the sclera in a longitudinal-9 orientation. The cutter was then slowly introduced through the trocar, directed toward the center of the eye, until a bright echo was visualized, accompanied by subtle posterior reverberation and mild posterior shadowing over the scleral tissue and periorbital fat. A characteristic double-line or “train-track” pattern can be observed, which undulates synchronously with the blade’s movements, along with the dynamic motion of the vitreous around the cutter’s tip. The cutter was held in position until no further vitreous movement was observed, at which point the surgeon proceeded to change location, always moving the probe and the cutter synchronously [4]. Then, the surgical microscope and posterior visualization system were set up again, and a second set of three still frames of the posterior pole under direct visualization was taken, with the same parameters employed at baseline. From that point onward, the surgery proceeded under direct visualization to eliminate any remaining vitreous hemorrhage, assess and repair any iatrogenic retinal breaks, and complete a full panretinal endolaser. The presence of suspected retinal touches, breaks, active retinal bleeding, or retinal proliferations unnoticed by the baseline ultrasound was carefully registered in the surgical log by a resident or fellow (adverse events). The timing of the time elapsed between the insertion of the first trocar and the end of the ultrasound-guided surgery (USG-PPV surgical time) was chronometered and recorded directly from the surgical video.

**Fig. 2:**
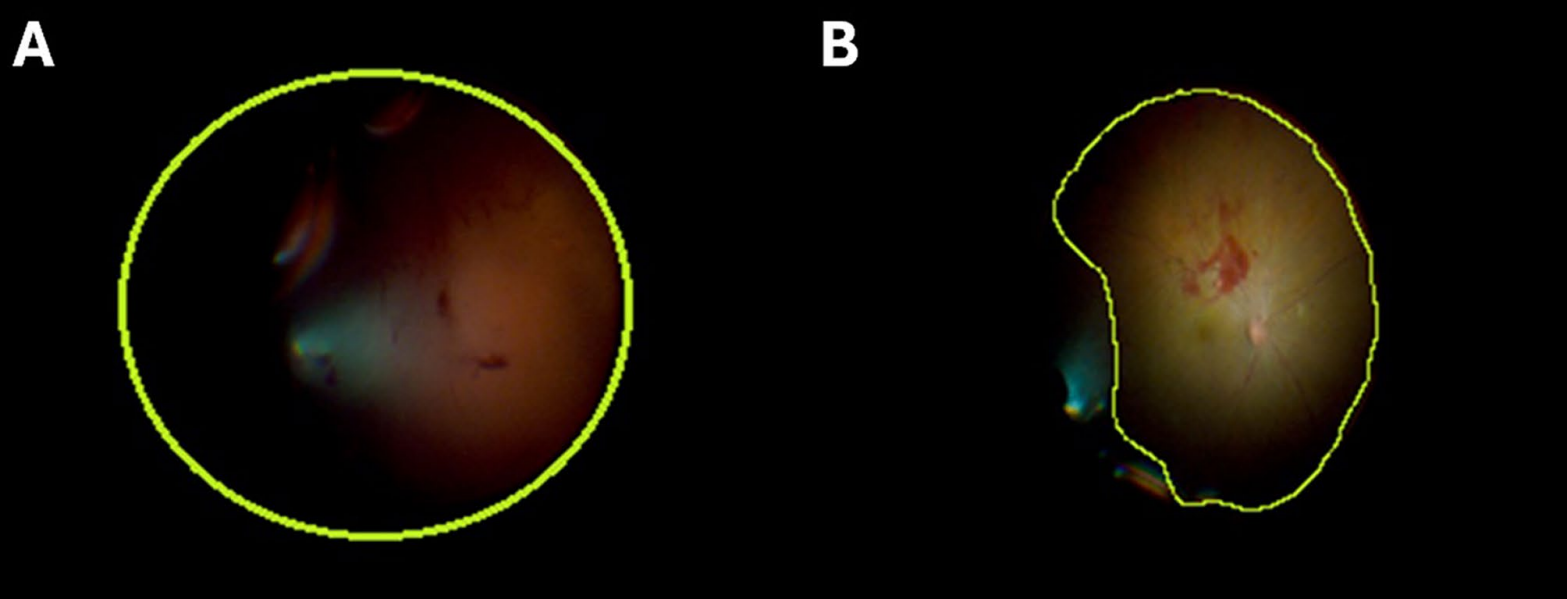
Digital segmentation and analysis of intraoperative images before and after the surgical technique. **A** shows the before and **B** the after

The surgical video and still frames from each participant were then downloaded onto a memory stick. The video was rewatched by the surgeon on a separate computer for a secondary assessment of surgical complications. The still frames were used for the digital estimation of the amount of eliminated vitreous hemorrhage. For this purpose, we used the open-source software ImageJ (National Institutes of Health, Bethesda, Maryland, USA; https://imagej.net/ij/; version IJ1). For the baseline still frames, the automatic segmentation tool on a black background was used to count the total number of pixels observed with the greatest saturation within the boundaries of an oval shape, centered on the surgical visual field and its circumference extending onto the edge of the surgical field. This total number of pixels represented the amount of vitreous hemorrhages contained in the observable vitreous cavity. We then divided the visual field into quadrants, and with manual segmentation (polygon selection tool), we delimited the areas of vitreous hemorrhages and the areas of observable retina free of hemorrhage. The entire process was then repeated with the second set of still frames (Fig. 2). The assessment of the baseline still frames and the second set of still frames was performed by two different teams of observers, who were blinded to the surgical video and to the unassigned set of still frames. All the measurements were performed in triplicate.

The proportions of vitreous hemorrhage and observable retina free of hemorrhage were calculated via the following formula:

$$\:\left(\frac{\sum\left(ms\right)}{{n}_{m}}\right)/\left(\frac{\sum\left(as\right)}{{n}_{a}}\right)*\left(100\right)$$

where *ms* is the number of counted pixels with manual segmentation in each quadrant, *as* is the counted pixels with automatic segmentation, *n*_m_ is the number of manual observations, and *n*_a_ is the number of automatic observations.

Statistical analysis was performed via an Excel spreadsheet (Excel 2010; Microsoft Corp., Redmond, WA) with XLSTAT v18.06 (Addinsoft, New York, NY). The general demographic data are presented as the means and proportions with standard deviations (SDs) and standard errors of the means (SEMs) when appropriate. The BCVA was converted into its logarithm of the minimum angle of resolution (logMAR) equivalent for statistical purposes. The visual acuity of counting fingers (CF) was 1.7 logMAR, that of hand movement (HM) was 2.0 logMAR, and that of light perception (LP) was 2.3 logMAR [9, 10]. Changes in BCVA and the number of counted pixels between groups were assessed via Student’s t-test, with an alpha value of 0.05 indicating statistical significance. The Gaussian distribution of all variables was determined via the D’Agostino–Pearson omnibus normality test. Interobserver agreement within each group of observers was assessed with a Cohen-Kappa test ± confidence intervals. A learning curve was plotted via the formula *y = ax^b.*

## Results

We enrolled 62 patients with a mean age of 64.3 ± 13.3 years. 55% were female, and 71% of the study eyes were left eyes. The mean USG-PPV surgical time was 13.5 min (range: 9.5–22 min). The mean BCVA at baseline was 2.05 ± 0.6 logMAR, and it was 0.58 ± 0.6 logMAR at 1 month of follow-up (*p* < 0.01). The mean letter gain after the surgery was 60 ± 12 letters.

**Fig. 3:**
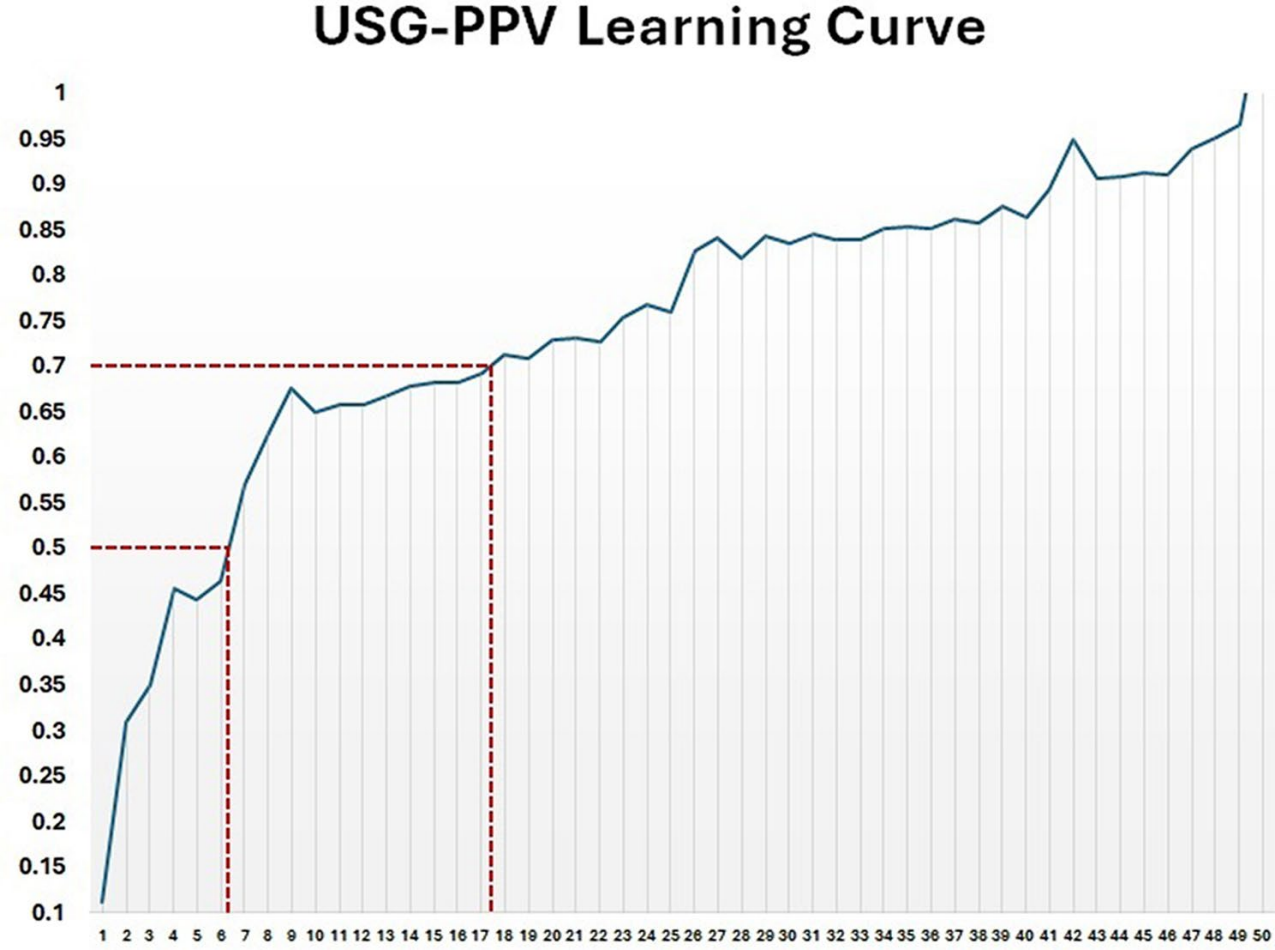
Experienced surgeon’s learning curve. The curve shows the minimum number of cases needed to effectively remove 50% or more and 70% or more visible vitreous. The curve shows that after 34 cases, the amount of vitreous removed could range between 85–90%

We had a total of 372 still frames, from which 1,104 individual pixel counts were performed with ImageJ software. Twelve images could not be processed due to corrupted files. Table 1 summarizes the pixel counts and their corresponding proportions of the vitreous hemorrhage and the observable retina free of hemorrhage before and after USG-PPV (baseline vs. second set of still frames). There was moderate interobserver agreement in the pixel counts among the members of each observer team (Baseline still frames: Cohen kappa: 0.52–0.58; second set of still frames: Cohen kappa: 0.44–0.51). There was a significant reduction in the number of pixels representing vitreous hemorrhage and a corresponding increase in the pixel count representing the observable retina free of hemorrhage after USG-PPV. Overall, ≈ 70% of the observable vitreous hemorrhage was removed (*p* < 0.01). Figure 3 shows the learning curve of the surgical technique. The minimum number of cases needed to achieve 50% or greater removal of the vitreous hemorrhage was 7. The minimum number of cases needed to achieve 70% or greater removal of the vitreous hemorrhage was 18.

**Table 1:** Changes in the pixel counts and corresponding proportion before and after the surgical technique. The table shows the change in visual acuity at the one-month follow-up visit. *BCVA: Best-corrected visual acuity. USG-PPV: Ultrasound-guided Pars plana vitrectomy

Assessment	Baseline	1 month	Delta	Alpha
BCVA* (logMAR)	HM	20/70	+ 60 letters	< 0.01
**Assessment**	**Before USG-PPV**	**After USG-PPB**	**Delta**	**Alpha**
Visible Vitreous Hemorrhage				
Pixels	427,694.4 ± 23,886	68,252.08 ± 76,275	-359,439.01	< 0.01
Proportion	88.6 ± 9.2%	16.9 ± 17.8%	-71.08%	< 0.01
Hemorrhage-free Retina				
Pixels	43,932.88 ± 41,289.8	394,906.1 ± 25,577.5	350,9732	< 0.01
Proportion	11.02 ± 9.4%	82.47 ± 17.7%	71.4%	< 0.01

Table 2 summarizes the observed adverse events (AEs) after USG-PPV. There were 11 AEs, two of which were serious, but only one was considered potentially related to the surgical technique. Posterior capsule ruptures occurred during phacoemulsification before the insertion of the trocars. The cases of optic nerve bleeding were mild and secondary to optic disc neovascularization due to uncontrolled diabetic retinopathy. All three instances stopped after the surgeon raised the intraocular pressure to 60 mmHg for 60 s. The cases of recurrence of hemorrhage in the vitreous cavity resolved spontaneously after two weeks without the need for further treatment and were considered to be due to poor diabetic control.

**Table 2:** Complications and adverse effects were recorded from the surgical log and the video review of the entire surgery by the main surgeon. The overall rate of adverse effects ranges from 1.6–4.8%. Y: yes. N: no. CI: confidence interval. *Postop: postoperative

Averse Effect	No. Cases	Related (Y/*N*)	Serious (Y/*N*)	Proportion (95%CI)
Posterior Capsule Rupture	2	N	N	3.23% (0.39–11.17%)
Optic Nerve Hemorrhage	3	N	N	4.8% (1.01–13.5%)
Vitreous Hemorrhage Postop*	4	N	N	6.4% (1.79–15.7%)
Retinal Detachment	1	N	Y	1.6% (0.04–8.66%)
Fibrinoid Reaction	1	Y	Y	1.6% (0.04–8.66%)

One case of retinal detachment occurred during the first month of follow-up. This serious AE was classified as not related to the USG-PPV because it occurred due to proliferative vitreoretinopathy from an iatrogenic nasal retinotomy that the surgeon performed under direct visualization while attempting to release traction from a very flat and stiff retinal proliferation that went unnoticed on the screening b-scan ultrasound. It was a macula-on detachment that was successfully treated with air-fluid exchange and silicone oil tamponade one month after the first surgery. The final BCVA was 20/40.

There was one case of extreme fibrinoid reaction in the anterior chamber that was classified as potentially related to the surgical technique. The patient was a 62-year-old diabetic female who confirmed no pain 24 h after surgery, with a BCVA of hand movements, + 4 cells in the anterior chamber, + 2 flares, abundant fibrin deposits in the pupil and iris, no hypopyon, and a normal conjunctival appearance. The surgical log from her surgery the previous day was uneventful. A b-scan ultrasound on the same day revealed an attached retina with multiple mobile medium-to-low echogenicity lesions, with no membrane formations, that could be vitreous hemorrhage cells. The patient was treated with a steroid therapeutic challenge for four hours (1% prednisolone acetate, 1 drop every 30 min for 4 h). The result was a decrease in the number of anterior chamber cells from + 4 to + 2. Therefore, the patient was administered a periocular injection of 5 mg betamethasone and observed for 24 h. The episode resolved with no further treatment 7 days after diagnosis. Vitreous and aqueous humor taps were performed, and the cultures were negative.

## Discussion

The surgical treatment of retinal diseases has significantly changed over the last three decades. During this time, we witnessed the abandonment of 20-gauge vitrectomy in favor of microincision vitrectomy; [11, 12] the introduction of novel surgical adjuvants such as silicon oils [13, 14], perfluorocarbon liquids [15], and vital dyes; [16] the commercialization of new and improved vitrectomy machines; [15, 17] and the development of surgical techniques that push the limits of surgeons, which has required them to adapt and develop new skills [18–20]. The result has been an unprecedented improvement in surgical outcomes. Pathologies that were once considered untreatable or to carry an almost certain prognosis of visual disability are now treatable and have visual prognoses similar to those observed in other surgical procedures, such as phacoemulsification [18, 21]. However, despite these advancements, one aspect that has remained unchanged since the beginning of modern retina surgery is the heavy reliance on optical equipment to perform surgery [22, 23]. Surgeons strive to ensure the best possible visualization of the surgical field to achieve the surgical goal. Overall, this means the removal or bypass of whatever is blocking the surgeons’ view, which usually takes the form of poor mydriasis or corneal, lens, aqueous, or vitreous opacities (or any variant or combination of the latter). Cases with poor visualization of intraocular structures during surgery are synonymous with greater difficulty, call for a more experienced and skillful surgeon, and are associated with poorer prognosis, largely due to a greater risk of iatrogenic retinal lesions [24, 25, 28]. Evidence has shown that, in the presence of dense vitreous hemorrhage or severe asteroid hialosis, the prevalence of retinal breaks secondary to poor visualization of the vitreous cutter may range from 8.1–76.92%.^24,25,28^ In the case of a blind procedure, the outcome is often less favorable than usual due to the urgency of the clinical circumstances and the inability to achieve crucial surgical goals. Moreover, the effectiveness of the procedure in removing the vitreous and the rate/risk of surgical complications during or after a blind procedure remain unknown [4]. 

In present times, in the case of an unavoidable corneal opacity, retina surgeons have a few options at their disposal before considering a blind vitrectomy procedure. In this context, a transient keratoprosthesis may be the best alternative in patients with severe corneal opacities [29]. It provides direct stereoscopic visualization of the posterior pole and peripheral retina. When properly placed, it creates a tight seal and provides a closed system for bimanual surgery [29]. Nevertheless, this comes with a risk of corneal or corneal graft decompensation that could range between 21% and 75%, postoperative glaucoma, and expulsive hemorrhages [30–32]. Moreover, in the presence of a severe corneal infection with scleral melting or scleral abscess, fixation of the keratoprosthesis could be extremely difficult or contraindicated [4]. The use of an ophthalmic endoscopic system could be another good alternative with several advantages as well. This device allows for an earlier procedure without requiring large corneal wounds, such as those associated with a transient keratoprosthesis [7, 8]. It provides excellent visualization of the space between the vitreous base and the posterior surface of the iris. Its lateral approach provides an unobstructed view, without being affected by the optical aberrations of the intraocular media [7, 8]. Its disadvantages are the relatively narrow field of view, lack of stereopsis, the impossibility of performing a bimanual technique, and hand-to-image dissociation [33]. Another drawback is that the camera and light source share the same equipment shaft. As a result, fogging and glare caused by nearby, dense vitreous opacities can occasionally obscure intraocular visualization, increasing the risk for iatrogenic injury [4]. Nevertheless, the most significant barriers to the widespread use of transient keratoprosthesis or ophthalmic endoscope, aside from the need for specially trained surgeons, are the lack of their availability and the high costs associated with their use [34, 35]. 

In the current manuscript, our group explores the efficacy and safety of a novel surgical technique that does not require the aid of optical equipment to achieve its goals. Our results show that USG-PPV can eliminate ≈ 70% of the vitreous without incurring major safety issues (potentially related adverse events: 1.6%, 95% CI: 0.04–8.66). Aside from one case of retinal detachment, which was classified as unrelated to the procedure, the most severe adverse event was a fibrinoid reaction. In this instance, the inflammatory response was primarily confined to the anterior chamber, with no pain, no conjunctival hyperemia beyond what is typically expected postoperatively, no hypopyon, had negative cultures, and a favorable clinical response to steroids. The reasons for this occurrence remain unclear. However, beyond the known patient-specific risk factors, such as uncontrolled type 2 diabetes, extensive panretinal photocoagulation during surgery, and the inherent proinflammatory state of the diabetic vitreous cavity [36], it is conceivable that a caustic substance, originating either from the latex glove or the surface of the ultrasound probe, may have entered the anterior or posterior chamber. This could have further compromised the blood-aqueous barrier and triggered a toxic inflammatory response [36, 37]. The learning curve for an experienced surgeon is manageable, the overall surgical time of the blind procedure is short, and the equipment required for its execution is inexpensive and readily accessible if not already available in most ophthalmology departments. Although no direct operational cost comparison is available among the techniques, data from third-party insurers (Medicare) and the UK’s National Health Service estimate that, even when using a reusable endoscopic probe, each procedure may cost between USD ≈ 1,088.27 and USD ≈ 5,809. This estimate excludes hospital fees and the cost of corneal tissue in cases involving a transient keratoprosthesis and is significantly higher than the approximate USD ≈ 200 cost of an ocular ultrasound [34, 35]. These factors make USG-PPV a potential alternative before considering a blind procedure or deferring the case. The technique offers the possibility to visualize major intraocular structures in real-time, such as the scleral wall, retina, and posterior lens capsule, along with the vitrectomy cutter. The result is an increase in localization awareness experienced by the surgeon, which improves the precision and safety of the intraocular instrument movements. Nevertheless, the resolution and 2D imaging are inferior to those provided by other visualization methods, such as a keratoprosthesis or ophthalmic endoscope. The hand-to-image dissociation is significant and influenced not only by the 2D image but also by the changes in orientation of the USB probe. Finally, the pressure exerted and manipulation of the ultrasound probe over the ocular surface can potentially induce corneal and conjunctival injury and may increase the risk of surface infections during the postoperative period, particularly if proper preparation is not undertaken.

Our study has several limitations that we would like to acknowledge. Although there is no precise method to quantify the vitreous gel or its volume in vivo, we used image analysis and pixel counting to obtain a reasonable approximation of what was possible to remove from the visible vitreous on a 2D image. Our methodology, although unique, is supported by previous studies that used the same software to digitally estimate other fluids, such as residual silicon oil after oil removal surgery [38]. This software tool has proven to be invaluable for the quantitative evaluation of visual structures. Its effectiveness in discriminating pixels enables it to accurately estimate areas and densities, as well as conduct morphological analysis of complex structures. In ophthalmology, ImageJ has been successfully utilized for analyzing endothelial cell growth and estimating vascular displacement after epiretinal membrane surgery [39, 40]. However, at the time this study was conducted, there was no specifically validated protocol or software plugin for accurate vitreous volume estimation. Even though we used a combination of automatic segmentation and manual correction to delineate the boundaries of the observable vitreous using the software’s built-in tools. The constant repetition and the existence of multiple observers may have led to the introduction of human error and observer bias. Moreover, the vitreous cavity is a 3D structure whose shape is similar but not exactly spherical. Readers should be aware that the numbers reported here are estimations and that the exact amount of eliminated vitreous with USG-PPV may vary and can be influenced by other factors that were not accounted for in this study (surgeon’s skill level and familiarity with the technique, vitrectomy machine parameters, dominant hand of the surgeon, the relative density of the vitreous hemorrhage and its time of evolution, the preexistence of posterior vitreous detachment, etc.). Another important issue to acknowledge is that the described learning curve was influenced by the previous experience of the main surgeon (RVM) [4]. The surgeon also served as the lead surgeon during the initial description of the USG-PPV technique, having performed 13 procedures before the start of this study. Consequently, the learning curve reported herein may be skewed to the right, with a more accurate estimate requiring approximately 20 procedures for ≥ 50% vitreous removal and around 31 procedures for ≥ 70% removal.

Finally, it is important to note that, although the rate of adverse events was very low, there is an inherent selection bias. This originates from the deliberate choice to perform the technique exclusively in pseudophakic patients, thereby eliminating the risk of iatrogenic lens damage, an ever-present possibility in a truly blind vitrectomy [41]. Moreover, the patient’s follow-up did not include the use of vital dyes such as lissamine green or fluorescein to assess corneal damage. Therefore, minor ocular surface trauma resulting from the use of the ultrasound probe may have gone undetected.

In summary, USG-PPV is a feasible technique that allows trained surgeons to remove ≈ 70% of the vitreous without the need for direct visualization; its safety profile is similar to that of vitrectomy cases with minimal visibility, and it could be considered a viable option in the absence of a transient keratoprosthesis or ophthalmic endoscope, especially in cases of extreme urgency.

## Supplementary Information

Below is the link to the electronic supplementary material.

Supplementary Material 1: Supplemental video 1 The video shows the preparation of the ultrasound probe, the general setting of the surgical field, and the ultrasound image with its corresponding movement from the outside, as well as the before and after ultrasound images of a case

Supplementary Material 2: Supplemental video 2 The video highlights the main surgical steps of the technique. The reader can observe how the vitreous moves around the cutter handpiece, the elimination of a dens subhyaloid hemorrhage, and the before and after comparison of several cases

## Data Availability

No datasets were generated or analysed during the current study.
